# DNA-nanostructure-assembly by sequential spotting

**DOI:** 10.1186/1477-3155-9-54

**Published:** 2011-11-18

**Authors:** Michael Breitenstein, Peter E Nielsen, Ralph Hölzel, Frank F Bier

**Affiliations:** 1 Fraunhofer Institute for Biomedical Engineering Department of Nanobiotechnology and Nanomedicine Am Mühlenberg 13, 14476 Potsdam, Germany; 2 University of Potsdam Institute for Biochemistry and Biology Karl-Liebknecht-Str. 24-25, 14476 Potsdam, Germany; 3 Department of Cellular and Molecular Medicine, Health Science Faculty University of Copenhagen Blegdamsvej 3c, DK-2100 N, Copenhagen, Denmark

## Abstract

**Background:**

The ability to create nanostructures with biomolecules is one of the key elements in nanobiotechnology. One of the problems is the expensive and mostly custom made equipment which is needed for their development. We intended to reduce material costs and aimed at miniaturization of the necessary tools that are essential for nanofabrication. Thus we combined the capabilities of molecular ink lithography with DNA-self-assembling capabilities to arrange DNA in an independent array which allows addressing molecules in nanoscale dimensions.

**Results:**

For the construction of DNA based nanostructures a method is presented that allows an arrangement of DNA strands in such a way that they can form a grid that only depends on the spotted pattern of the anchor molecules. An atomic force microscope (AFM) has been used for molecular ink lithography to generate small spots. The sequential spotting process allows the immobilization of several different functional biomolecules with a single AFM-tip. This grid which delivers specific addresses for the prepared DNA-strand serves as a two-dimensional anchor to arrange the sequence according to the pattern. Once the DNA-nanoarray has been formed, it can be functionalized by PNA (peptide nucleic acid) to incorporate advanced structures.

**Conclusions:**

The production of DNA-nanoarrays is a promising task for nanobiotechnology. The described method allows convenient and low cost preparation of nanoarrays. PNA can be used for complex functionalization purposes as well as a structural element.

## Background

The construction of nanostructures is a challenging and resource intensive task for biotechnology. The two classical ways are the top-down and bottom-up approaches. The best known top-down method was introduced in 1999 by Mirkin et al. [1-4] where an atomic force microscope tip was used for direct writing a chemically active ink on a gold-surface. The achieved method is now known as Dip-Pen-Nanolithography and is capable of generating feature sizes in the range of 50 nm [5,6]. Further methods that aim on the preparation of nanostructures employ electrochemical deposition of metal salts [7], use of photomasks [8] or use of nanoscale stamps [9]. But all these methods are cost intensive and very challenging in terms of applying more than one spotting component. On the other hand, in the bottom-up approach the self-assembly capabilities of biomolecules like DNA are exploited to the design of nanostructures. Up to now, many promising ways have been published for DNA [10-13] and even RNA-structures [14] to achieve molecular sized structures. But predominantly such complex DNA-structures are only randomly fixed on a surface without a defined position.

Here we present a simple method that combines top-down and bottom-up approaches to generate a DNA-based nanostructure. This toolbox uses the high flexibility of an atomic force microscope which is one of the most powerful mechanical tools in nanotechnology. The key elements of this device are the piezoelectric actuators, enabling nanoscopic small movements with high precision. In our approach the top-down tool is combined with the precision of bottom-up DNA base pairing. The simple building rules of DNA-base-pairing are being used for the creation of more complex nanostructures that easily skip the limitation of all mechanically based top-down techniques. The key element is to create sophisticated structures by hierarchical assembly. In addition we employ PNA (peptide nucleic acid [15,16]) as a powerful tool to extend the DNA's building capabilities and to functionalize existing DNA strands with junctions.

## Results and Discussion

The preparation of DNA nanostructures on a solid support is based on a recently developed method [17] in our group that provides fixed nanoscale anchors on a surface. Therefore we immobilized biomolecules like DNA-oligonucleotides on a functionalized glass support by using general AFM-techniques. This method facilitates the deposition of different biotinylated biomolecules by a single AFM-tip without the need to optimize spotting conditions for each substance. Only a single ink, namely neutravidin in glycerol, is used for spotting, which reduces technical requirements and can therefore be easily adapted to most AFMs. Neutravidin is used as the linking element because of its capability for binding up to four biotin molecules. Consequently this enables the immobilization of any biotinylated biomolecule on that specific neutravidin spot by AFM deposition.

**Figure 1:**
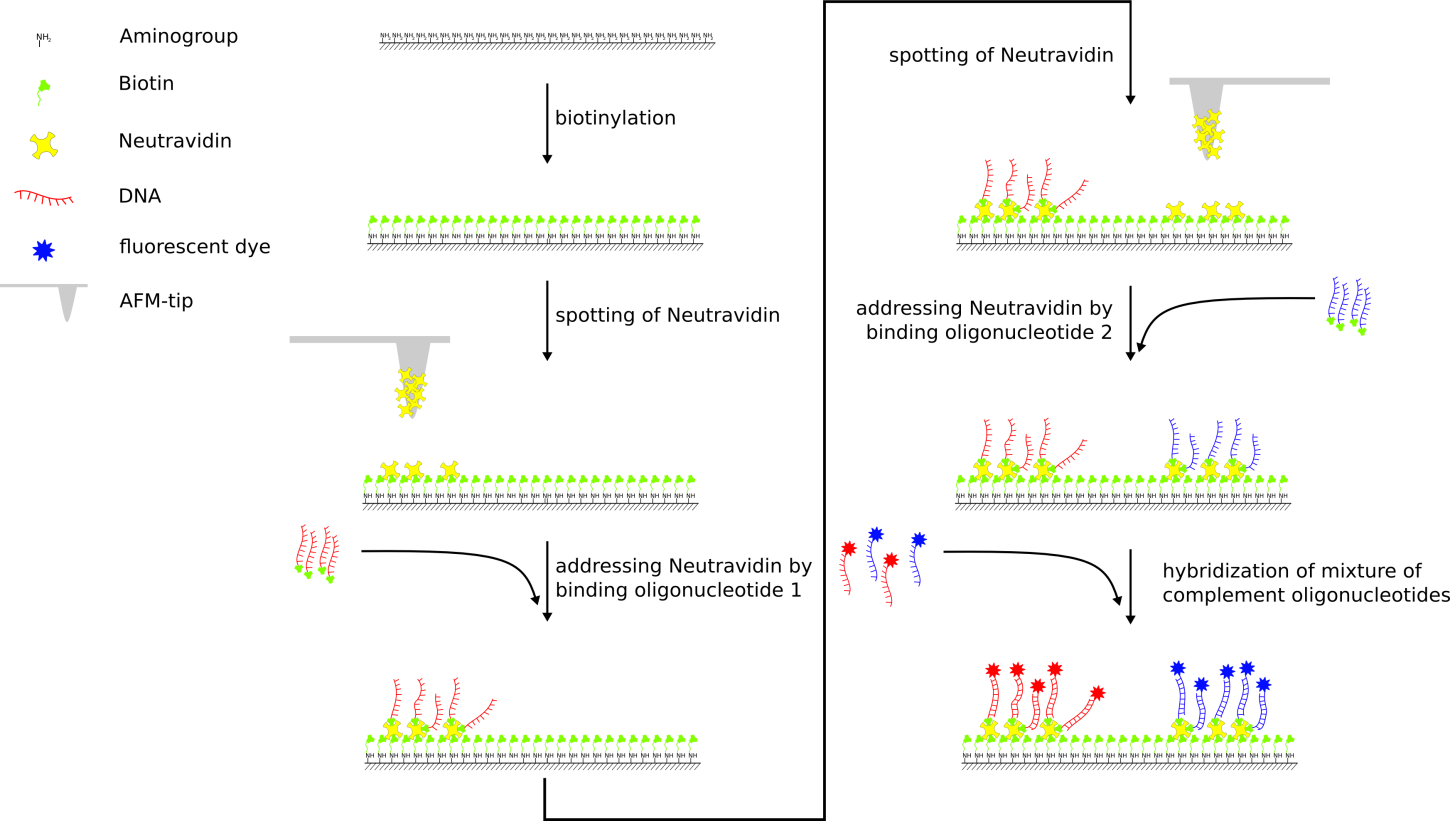
**Overview of the spotting method**. Spotting is carried out on a biotin functionalized glass surface. Neutravidin is spotted as a linking element, followed by binding of a biotin functionalized oligonucleotide. Further spots and addressing can be added by repeated spotting without replacing the surface. The cycle of spotting and binding can be repeated several times to compile the array [17].

**Figure 2:**
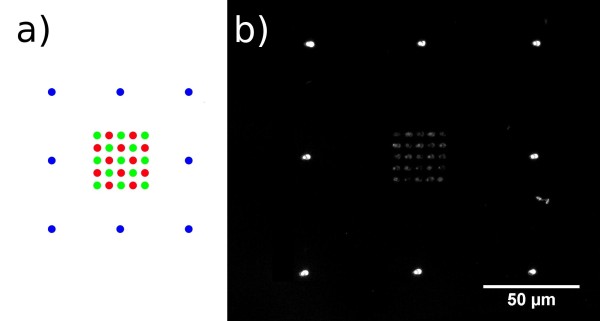
**Spotting scheme of the sequential process**. a) Anchor for the right handed DNA-construct represented by green dots, complement of the array with the anchor for the left handed DNA-construct with red dots, together with the ancillary frame for a convenient workflow in blue dots. b) Fluorescence microscopic image of the whole array with the ancillary frame and the region of interest by fluorescent excitation of the green fluorescent dye DY547.

The sequential spotting takes place on a biotinylated glass surface. The process is illustrated in figure 1 and shows that after each neutravidin spotting an incubation with address-molecules which are aimed to be immobilized follows the spotting of each sub-array. Once the neutravidin binding sites have been occupied and all spots are saturated, the sequential spotting can be repeated to complete the process for all undetermined positions. In the first round all neutravidin spots are incubated with a biotinylated oligonucleotide (LcF5 - red dots in figure 2a) which is capable of binding the site A of the DNA-construct. In the following round the second oligonucleotide (RcF6 - green dots in figure 2a), complementary to side B of the DNA-construct, is attached. For localizing and visualizing the position of the array during fluorescence microscopic investigation, we used the green fluorescent dye DY547 (blue dots in figure 1) which was spotted in the third and final round. The frame with its own dye and excitation wavelength has the function to minimize light exposure in the cource of localizing and focussing on the DNA-nanostructure and consequently minimizes bleaching of the actual region of interest.

**Figure 3:**
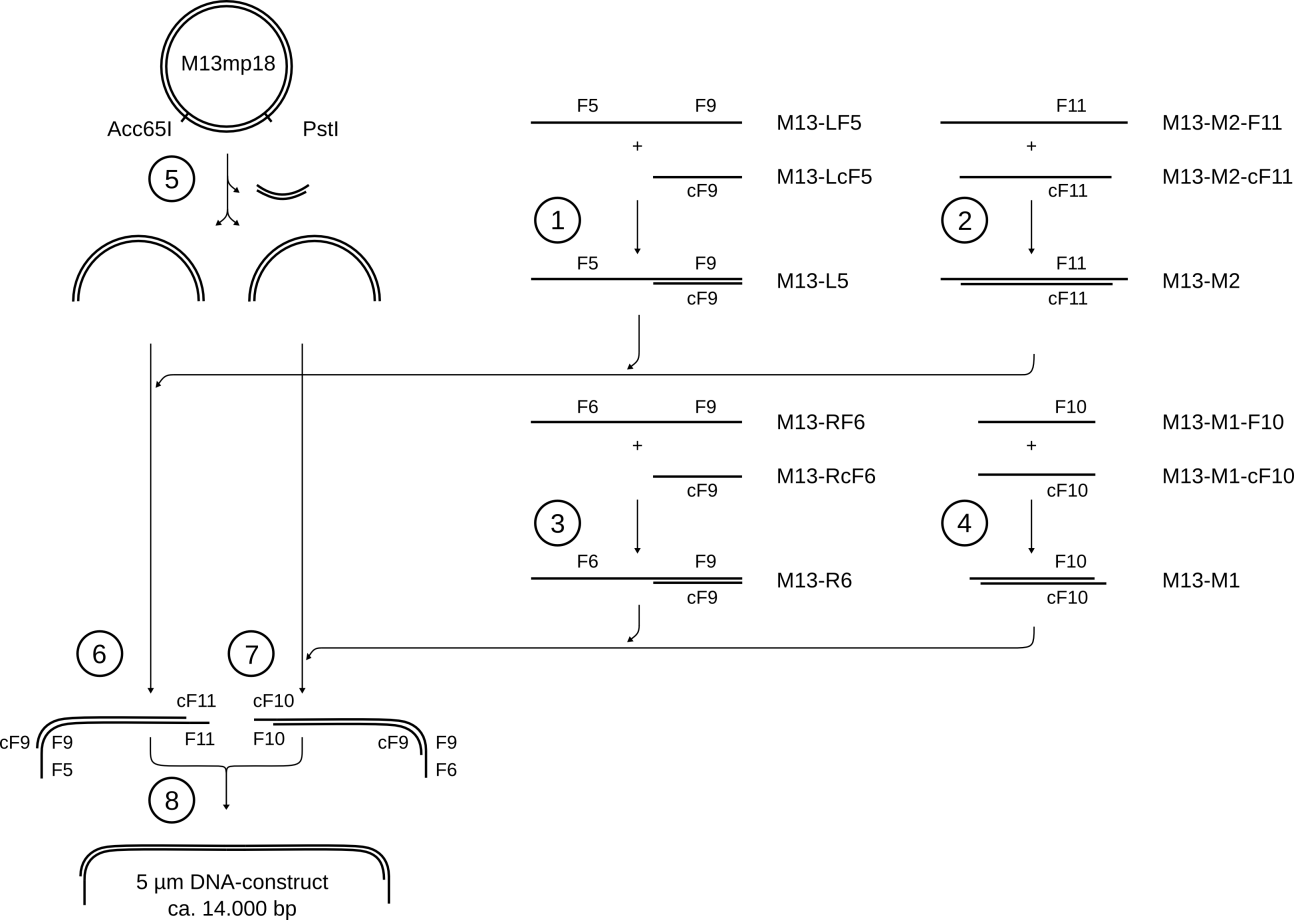
**DNA-construct assembly pathway**. The vector M13mp18 (7250 bp) is used to produce a DNA building unit of approximately 5 μm length with an elongated single stranded sticky end at both sides. 1 - 4: Formation of single stranded adapter oligonucleotides. 6, 7: Ligation of adapter elements to form two DNA-constructs that are capable to bind site specific. 8: Hybridization will occur spontaneously and will result in the long DNA-construct.

In the second step of the nanostructure construction, the top-down approach of spotting was combined with the bottom-up methodology, which is based on DNA hybridization with the oligonucleotide on the surface. It spontaneously takes place as soon as the DNA-construct is loaded within the spotted array. Figure 3 illustrates the pathway for the preparation of the necessary 5 μm long double stranded DNA building element (for detailed sequences see additional file 1). With different sticky ends on both sides, allowing site directed hybridization, the orientation of the DNA strand can be defined.

**Figure 4:**
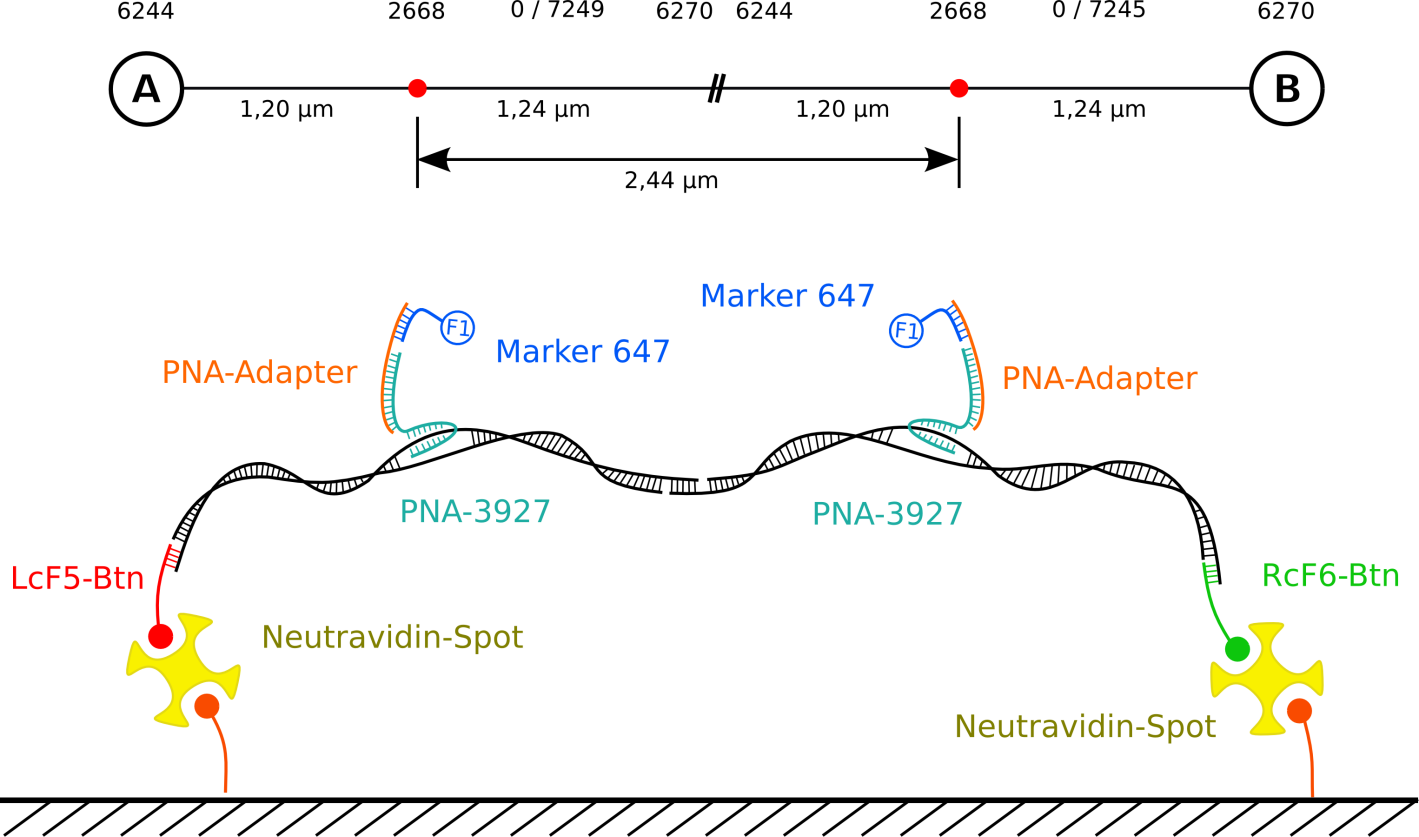
**Spanned DNA-Nanostructure**. The construct is site directed immobilized via biotin and neutravidin based linking elements and can be combined with a PNA-functionalization.

The scheme in figure 4 illustrates the construct and its dimension. The size of the construct from side A to B is 14442 bp (4.91 μm). From side A (LcF5) to the first PNA binding site it measures 3576 bp (1.20 μm) and from side B (RcF6) to the second PNA binding site 3643 bp (1.24 μm). Both PNA binding sites have a mutual distance of 7223 bp (2.44 μm).

**Figure 5:**
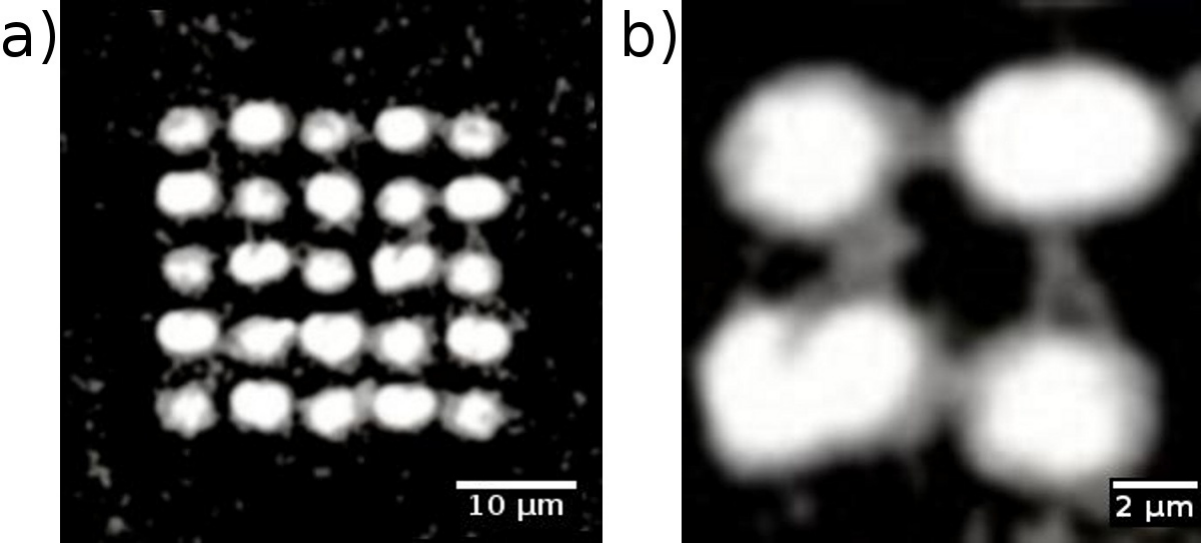
**Fluorescence microscopic image**. After array-preparation DNA-construct side A and B self-assembly was initiated by transferring a diluted mixture of side A and side B DNA-constructs directly onto the array. A complete DNA bridge has been formed that connects the spots. DNA is visualized by SYBR-Green I.

The nanostructuring process starts by generating a two-component array with spot-center distances of 5.8 μm. The spot-diameter varies between 2 and 3 μm, so that it results in a gap of about 2.8 - 3.8 μm. The described DNA-construct measures 4.9 μm and would be able to interact with the spotted anchors to bridge the gap between appropriate spots. The neutravidin spots are addressed by biotinylated oligonucleotides which are complementary to the DNA-construct. Each spot has its unique oligonucleotide-address. Initiation of the bottom-up part is induced by transferring the synthesized DNA-construct with its sticky ends generating the nanostructure. This happens within the prepared array according to the spotted pattern. To visualize the DNA, it was stained with SYBR-Green I and viewed by fluorescence microscopy. The resulting structure is shown in figure 5. The direct connection of the spots with DNA can clearly be seen as fine lines between the spots.

Double-stranded DNA is quite a rigid macromolecule with a persistence length of about 50 nm which is, compared to single-stranded DNA with 1 - 4 nm persistence length, rather stiff [18]. However, the use of dsDNA is advantageous not only because of its stability, but because of the possibility for protein induced and molecular recognition induced DNA-binding, as well as for PNA-interactions.

**Figure 6:**
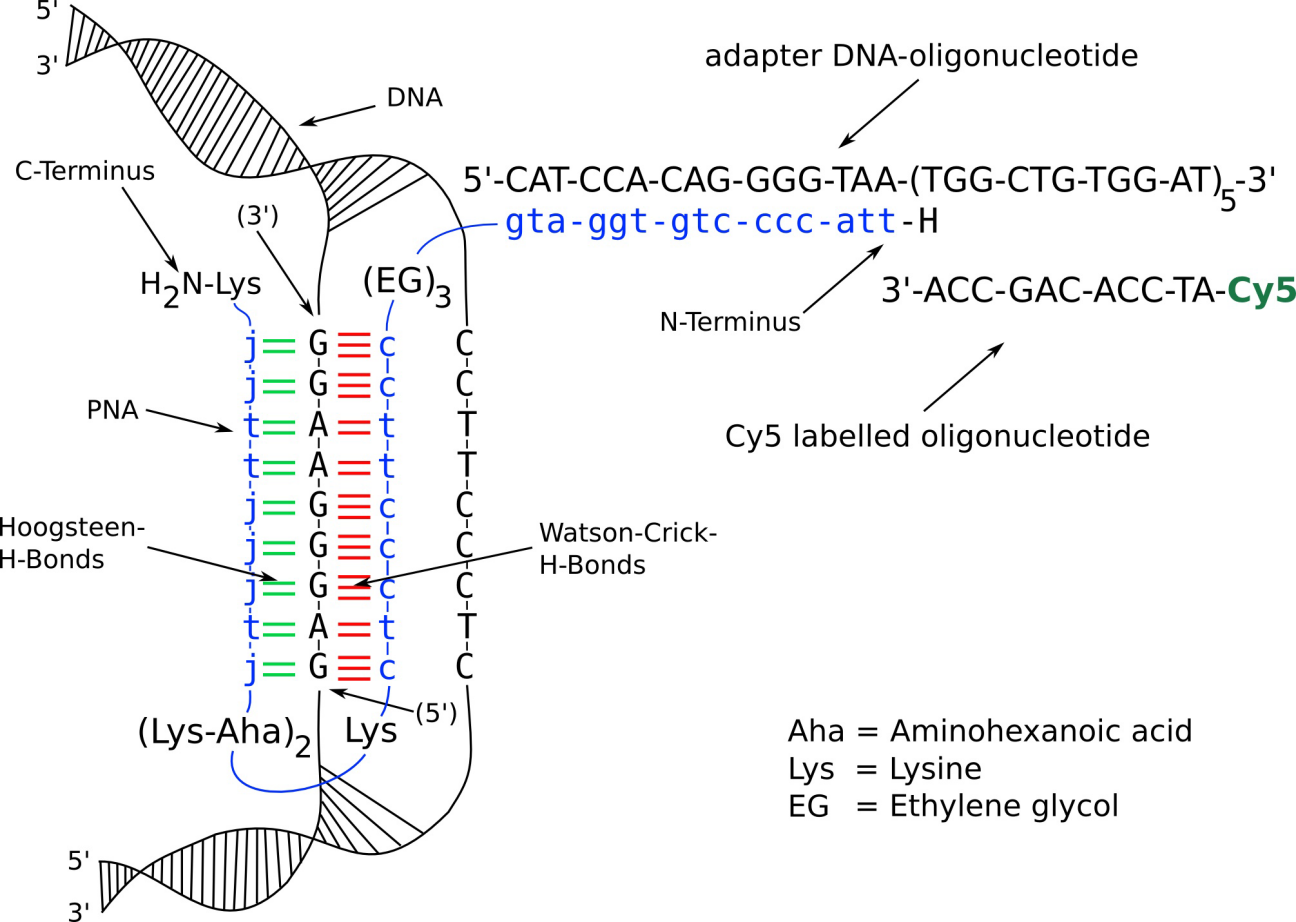
**Sequence of the homopurine DNA target**. PNA strand bound to double stranded DNA by forming a triplex invasion complex. For better solubility in water, lysine and ethylene glycol linkers were incorporated in the PNA strand. By substitution of cytosine by pseudoisocytosines (J-bases) the pH dependence of the binding process was shifted to a more neutral one. Through the elongated PNA-tail a DNA adapter oligonucleotide can be used for further functionalization purposes - here demonstrated on five repetitive and Cy5 labelled elements.

PNA is a structural DNA mimic where the phosphodiester backbone is replaced by a 2-aminoethyl-glycine polyamide (peptide) backbone. Consequently PNA can hybridize in the common way as it is known for DNA. Here it is used because of its triple complex binding capability where it forms an invasion complex at homopurine DNA targets [19-21]. The strand invasion and triple helix formation ability of PNA is a powerful tool in terms of using PNA as a linking element as shown in figure 6. The M13mp18 vector, which is the building block of our processed construct, has a unique region that fulfils all requirements for the purpose of binding in terms of a homopurine sequence at the vector's position 2668. The sequence can be seen in figure 6 and also shows the assembly of our design. The PNA-3927 was constructed as a bis-PNA with replacement of the cytosines in the Hoogsteen strand by pseudoisocytosine (J-base), allowing efficient DNA triplex binding at neutral pH [21,22]. Furthermore, lysines were introduced in the linker between the two bis-PNA domains to increase binding efficiency [16], and a 15-mer PNA adaptor domain was attached to the N-terminal of the bis-PNA via a triple "ethylene glycol" (8-amino-3,6-dioxaoctanoic acid) (EG) linker.

To have a universal tool, a DNA-oligonucleotide as adapter element can bind to the clasping PNA. A second DNA-oligonucleotide which is labelled by a fluorescent dye was used for visualisation of the nanoconstruct under fluorescence microscopy conditions. The adapter oligonucleotide is equipped with five repetitive sequence patterns to bind up to five identical, fluorescently labelled oligonucleotides for fluorescence enhancement. Additionally, it is important to take into consideration that the triplex invasion binding of PNA to the duplex DNA is significantly favoured at low ionic strength [16], which strongly disfavour DNA-DNA hybridization. Thus the PNA should be bound to the DNA-construct at low ionic strength before all the following steps. Once the triplex has formed the ionic strength has no impact on the triplex stability and facilitates to change to any desired buffer [16].

**Figure 7:**
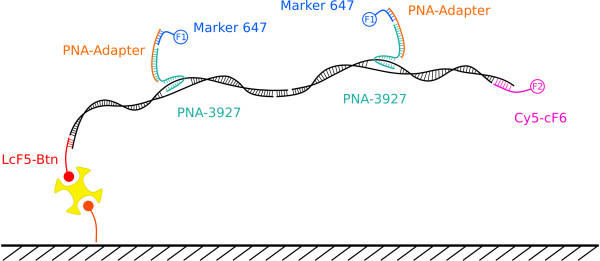
**PNA binding and combined DNA-orientation test**. The oligonucleotide LCF5-Btn attaches side A of the double DNA-construct to the surface and immobilizes the strand. On the side B a probe oligonucleotide (Cy5-cF6) which is Cy5 labelled is bound. The PNA-3927 can bind to the double stranded DNA at position 2668. Because two strands were combined, the PNA can bind to two identical positions. With help of an adapter oligonucleotide the PNA is detected by hybridizing a fluorescence labelled marker.

**Figure 8:**
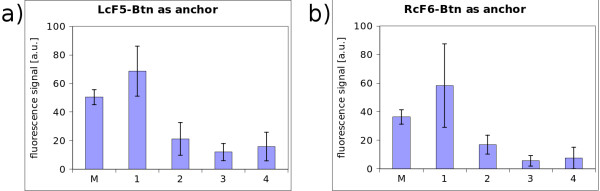
**Results of the microarray test**. PNA-binding test - 1: with all elements that are needed like LcF5-Btn, PNA-3927 and PNA-Adapter - 2: lacks PNA - 3: Adapter is missing - 4: neither PNA nor Adapter are in the test solution - M: marker as positive control. a) and b) have different anchors and opposite orientations.

To ensure that the synthesized PNA, which is a challenging long oligomer, binds at the appropriate position on the DNA, a conventional microarray test was carried out. To achieve the same chemical properties as they are needed for the nanostructural approach, the glass slides were silanized, biotinylated and functionalized with neutravidin. This is the same design as spotting with the AFM. Then several solutions were spotted conventionally with a microarray spotter. Figure 7 shows all the necessary elements: the PNA, the PNA-Adapter and the Cy5 labelled oligonucleotide as marker for visualization. The oligonucleotide LcF5-Btn binds to side A of the DNA-construct (see figure 7; LcF5 on side A; RcF6 on side B). The PNA-3927 forms a triplex-invasion complex on the DNA-construct while the adapter DNA can bind to the PNA and has five binding sites for the fluorescent DY-647 labelled probe. Figure 8a shows the results of the essential combinations: 1) LcF5-Btn together with PNA-3927 and PNA-adapter were incubated in a solution, containing the DNA-construct; 2) is similar to 1, but lacks PNA-3927; 3) is similar to 1 but has no PNA-Adapter and 4) only contains the DNA-construct and LcF5-Btn. The high fluorescence signal of the Marker in solution 1 is used as control experiment. In all the other variants at least one essential element for obtaining a specific fluorescence signal is missing. Thus it can be deduced that PNA binds to the DNA which is used for the nanostructure.

**Figure 9:**
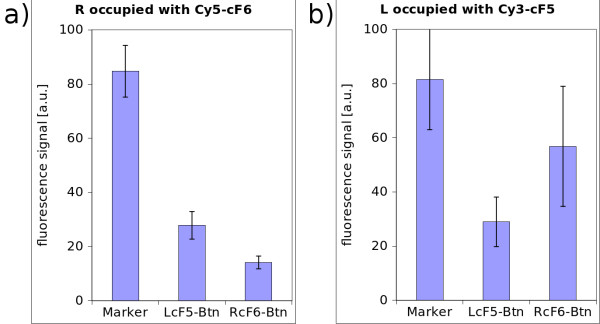
**Test for construct orientation**. a) L represents the successful binding situation shown in figure 8 - Cy5-cF6 was prehybridized whereas LcF5-Btn is located in the corresponding spot whose signal is plotted here - R: catcher oligonucleotides for the right side, which cannot bind to the DNA strand because of the prehybridization with Cy5-cF6 - b) the opposite end was prehybridized with Cy3-cF5 so that the whole ensemble should only bind in the opposite direction.

Inspecting figure 7 in detail, it shows that LcF5-Btn is bound at side A and the Cy5 labelled probe Cy5-cF6 is bound at side B of the double DNA construct. Both probes were incubated and hybridized to the DNA before they were applied on the microarray. From the sequences it follows that by changing the functional elements of these probes, the orientation of the whole construct can be turned around. By incubating with Cy3-cF5 which will hybridize on end A and by incubating RcF6-Btn, fitting on end B, a completely different signal should appear, because the fluorescence dyes Cy3 (532 nm excitation maximum) and Cy5 (635 nm excitation maximum) reveal the orientation. The setup was as follows: the same glass-microchip as described above, was used with immobilized LcF5-Btn and RcF6-Btn for testing the PNA binding capability. The chip provides appropriate anchors for either the fixed A or B side orientation of the DNA-construct. The result of incubating the DNA double construct with prehybridized Cy5-cF6 is shown in figure 9a. In this case the single stranded overlap on the right side of the DNA is occupied and cannot bind to those spots, which offers complementary binding sites. Only at those spots which offer complements for side A, the Cy5-cF6 hybridized strand can bind, and vice versa. The consequence is a significantly higher fluorescence signal as shown in figure 9a. In figure 9b the orientation is inverted by prehybridizing Cy3-cF5. In summary, this test reveals that the assembly of the DNA-double construct has been successful and that the single stranded sticky ends can indeed be addressed individually.

Furthermore, it shows that the orientation of the DNA-nanostructure in figure 5 is orientated according to the spotting pattern.

**Figure 10:**
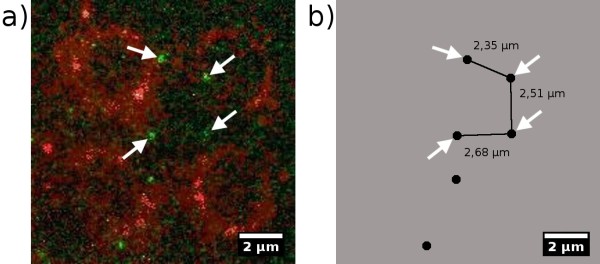
**Visualized PNA on DNA strands**. a) The fluorescently labelled PNA can be seen as green dots. Their mutual distance on the DNA-construct is 2.45 μm and they are located on the predicted positions on the DNA-strands. Red circles are the spots, serving as DNA-anchors. b) Four of the relative distances of the detected PNA-lables are highlighted. Their relative positions fit quite well to the theoretical predictions derived from the DNA-construct.

The result of the DNA-assembly together with PNA-nanofunctionalization is shown in figure 10. The edges of the spots can be observed as red rings (compare to figure 5). From this it follows that they are loaded with the biotinylated oligonucleotide (see figure 2). The additional binding to the spots can be explained by incomplete coverage of the biotin binding sites during incubation [17]. Consequently, the dye can bind to some remaining binding sites during the last incubation step in which the ancillary frame was spotted. The green dots between the spots represent the DY547 labelled PNA being bound to the DNA-construct between the spots. Two binding sites for PNA are available on each of both hybridized DNA-constructs (compare figure 4). Assuming a stretched double helix, a theoretical distance of 2.45 μm (7223 bp) can be reached. This agrees very well with the measured distance of 2.35 μm following from the fluorescence images, shown in figure 10. It should be taken into consideration that DNA normally is randomly coiled and needs to be extended in order to form such a structure. And neither any force, nor any directed flow was applied to the sample to assist arranging the DNA strand into a favourable direction. The very low DNA concentration used in this setup allows to obtain only a few directly DNA-connected anchor-spots.

## Conclusions

We have demonstrated a novel approach for using DNA as a building unit for surface-bound nanostructures. The structure does not depend on a fixed pattern and is immobilized at a defined position with well defined dimensions.

For generating such a structure only two key features are necessary: the first is a method that allows immobilization of different biomolecules, e.g. DNA oligonucleotides, on a surface with high spatial resolution. In this work we have shown that the sequential spotting method as illustrated in figure 1 fulfils this requirement. The second feature is a refined DNA-design that allows self-assembly of the nanostructure.

The prepared nanostructure also remains chemically accessible for subsequent biomolecular recognition such as by PNA. The PNA formed structure can be regarded as a versatile construction and linking element that facilitates the further building of complex superstructures. Binding PNA to dsDNA by triplex invasion has been tested and proved by microarray analysis and fluorescence microscopy.

The spotting-method itself is easy and does not require complex preparatory work. It has been designed with the aim to facilitate the employment of most atomic force microscopes. Therefore the presented method can be integrated readily into many nanotechnology applications and key questions. An upgrade of most AFMs is cogitable and thus is cost efficient because beside the common AFM-equipment or equivalent nanomanipulation tools only commonly available chemical compounds like biotinylated oligonucleotides and neutravidin in combination with DNA are required.

The outlook predominantly addresses the analysis of single molecule interactions. The investigation of RNA in single cells, for example, is limited by the faint concentration and might take advantage of structures on a molecular level. DNA based computing machines that are based on FRET (fluorescence resonance energy transfer), as described in [23], could benefit from the arrangement of the presented nanostructure and would result in a exciting combination of biology and electronic. We also have miniaturized array-technology together with micrufluidic point-of-care diagnostic approaches in our focus, which might lead us to lab-in-the-blood devices.

## Methods

### Silanization and biotinylation

Glass slides (Menzel Gläser, Menzel GmbH & Co. KG, 38116 Braunschweig, Germany) were cleaned with ultrasound in acetone for 15 minutes and again in ethanol (acetone and ethanol were obtained from Carl Roth GmbH & Co. KG, Karlsruhe, Germany). After rinsing with ultrapure water, the slides were put into NaOH (10 M) for 1 minute and washed thoroughly with water. Drying was carried out in a centrifuge (Varifuge 3.0R, Heraeus) for 1 minute. In the vapor phase at 120°C silanization with 3-Aminopropyltriethoxysilane (Fluka Chemie GmbH, 89552 Steinheim, Germany) was executed in a sealed beaker and finished after 60 minutes. For biotinylation, Sulfo-NHS-Biotin (20 mg) (Thermo Scientific, IL 61101 USA) was dissolved in DMSO (1 mL) (Carl Roth GmbH & Co. KG) because of its low stability and moisture-sensitivity. The DMSO solved Sulfo-NHS-Biotin can be stored at -20°C with desiccant.

Sulfo-NHS-Biotin (10 mL) solution was added to Na_2_HPO_4 _(100 mM, 21 mL), NaCl (150 mM) buffer at pH 7.4. Incubation of 5 silanized glass slides took place for 3 hours at room temperature. Slides were washed with PBS and rinsed with water. Blocking was carried out by incubating the glass slides in a freshly prepared, 0.1% (w/v) solution of blocking reagent CA from Applichem in 100 mM Tris-Cl. For cleaning, slides were washed three times for 5 minutes in Tris-Cl (100 mM Tris, 600 mM NaCl, pH 7.4) and finally rinsed with ultrapure water. NaOH, Na_2_HPO_4_, NaCl, PBS and blocking reagent CA were obtained from AppliChem GmbH, 64291 Dortmund, Germany.

### DNA-Array preparation

Neutravidin (Thermo Scientific, IL 61101 USA) that had been spotted had to be addressed by biotinylated oligonucleotides (Biomers.net GmbH, 89077 Ulm, Germany). Sequences of the oligonucleotides: Side A LcF5: 5'-CTT ATC GCT TTA TGA CCG GAC C-3' (5': Biotin); Side B RcF6: 5'-CAA TGA AAC ACT AGG CGA GGA C-3' (5': Biotin). Staining of the outer frame was done with biotinylated DY-547 dye (Dyomics GmbH, 67745 Jena, Germany). All these three components were diluted in carbonate buffer pH 9.0 to a final concentration of 1 mM. Incubation time for binding was 5 minutes and was stopped by washing with 1× PBS-buffer and ultrapure water. The left DNA strand M13-L part and right DNA M13-R strand were diluted 1:50 and 5 μl of each solution were transferred onto the chip directly to the prepared array. After incubation in the dark for 60 minutes in TE-buffer at 37°C and 85% rel. humidity, the glass chip was washed by completely dipping it into PBS-Tween and rinsing it a second time in PBS.

### Spotting

An atomic force microscope CP-II from Veeco (Santa Barbara CA, 93117 USA) and AFM-tips from NanoSensors (NanoAndMore GmbH, 35578 Wetzlar, Germany) were used: DT-CONTR (force constant: 0.2 N/m; resonance frequency: 13 kHz). Movement of the AFM-tip and execution were controlled by the diNanolithography Software V.1.8. Approaching the biotinylated glass slide was achieved in contact mode with 3.4 mN contact force. The tip remained in contact for 4 seconds and changed to the next spotting positions by retraction. Ink was supplied to the tip by a hypodermic needle of Popper & Sons, Inc. (N.Y. 11040 USA).

### DNA preparation

The DNA-construct was generated by digesting 10 μg M13mp18 RF I DNA plasmid (New England - BioLabs GmbH, 65926 Frankfurt a. M., Germany) simultaneously with the restriction enzymes PstI, Acc65I and BamHI (New England - BioLabs GmbH, 65926 Frankfurt a. M., Germany) in NEBuffer-3 at 37°C for 2 h. Then the enzymes were inactivated by heating the batch to 80°C for 20 minutes and finally cooling down slowly (1 K/min.). Parallel to this, hybridization of the adapter segments in Tris-Cl buffer (100 mM Tris-Cl; 600 mM NaCl; pH 7.4) took place by heating the oligonucleotides up to 90°C for 5 minutes (see figure 4) and cooling down slowly (1 K/min.). The digested M13mp18 plasmid (120 μl) was then divided into a left and right batch. The left was incubated with 8 μl M13-L5 (10 μM) and 8 μl M13-M2 (10 μM). The right was incubated with 8 μl M13-R6 (10 μM) and 8 μl M13-M1 (10 μM). Prehybridization took place for 30 minutes at 40°C, then 30 minutes at 30°C followed by cooling down to 20°C. Both batches were then ligated separately with T4 DNA ligase (New England - BioLabs GmbH, 65926 Frankfurt a. M., Germany) over night at 4°C. To avoid rupture of the sensitive construct, ligation was not stopped by heating but by removing the enzyme by cleaning it with Sure Clean (Bioline GmbH, 14943 Luckenwalde, Germany) and dissolving it in 100 μl TE-buffer (50 mM Tris-Cl, 100 mM NaCl). The concentration of both, the left and the right batch, were equalized by adding TE-buffer to a final concentration of about 30 ng/μl. The product was then stored at -20°C.

### PNA synthesis

The PNA 3927 was synthesized by conventional solid phase Boc chemistry as previously described [24,25], and purified by reversed phase HPLC. The PNA was subsequently characterized by HPLC and MALDI-TOF mass spectrometry (see additional file 2). Furthermore, the thermal stability (T_*m*_) of complexes with an oligonucleotide (5'-GAG GGA AGG-3') binding to the triplex domain and an ologonucleotide (5'-CAT CCA CAG GGG TAA-3') was determined as 87°C and 77°C, respectively (see additional file 3), showing that both domains are functional in terms of hybridization to a DNA target.

### Microarray test

Glass slides (Menzel Gläser, Menzel GmbH & Co. KG, 38116 Braunschweig, Germany) were blocked 1 h with 0.1% blocking reagent CA (AppliChem GmbH, 06466 Gatersleben, Germany) after they were silanized and biotinylated as described above. The reactive glass slides were incubated over night with 25 ng/ml Avidin at room temperature. Microarrays were spotted contactless with the microarray spotter TopSpot (BioFluidiX GmbH, 79110 Freiburg, Germany) on the functionalized and blocked glass slides. The solutions that have been spotted were: 2.7 μl left DNA construct, 2.7 μl right DNA construct, 2.7 μl PNA-adapter and either 2.7 μl LcF5-Btn 1 μm or 2.7 μl RcF6-Btn 1 μm. For a negative sample one component was omitted (PNA or adapter oligonucleotide). Incubation took place at 25°C at 85% rel. humidity for 1 h. For detecting the PNA and the DNA's orientation a Cy5 labelled oligonucleotide (Cy5-cF6; 1 μl) and a Cy3 labeled oligonucleotide (Cy3-cF5; 1 μl) were hybridized at 35°C at 85% rel. humidity for 1 h and were finally detected by a fluorescence microarray scanner (Axon Instruments, GenePix 4200A).

### Microscopy

Fluorescence microscopy was carried out with an upright epifluorescence microscope Olympus A BX51 (objective: UPlanFL N; 40 × 0.75). Fluorescence detection was accomplished with the following filter-cube combinations: DY-547 detection: excitation filter (Ex) BP 545/25, dichromatic mirror (Dm) 565, emission filter (Em) LP 605/70 and for SYBR-Green I detection: Ex BP 460 - 495, Dm 505, Em LP 510 - 550. For illumination a mercury arc lamp (100 W, OSRAM GmbH, 81543 München, Germany) in combination with a Uniblitz VCM-D1 shutter was used. Image acquisition was carried out with a CCD camera (FView II) with 12 bit dynamic range and 1376 × 1032 pixel resolution. Software aquisition was donw with cellˆR version 3.1 (build 1276). Image editing was realized with ImageJ V1.42q. Staining of DNA was performed with SYBR-Green I (1:10000 in DMSO).

## Competing interests

The authors declare that they have no competing interests.

## Authors' contributions

MB developed the sequential spotting method and all experimental setups and the design of the nanostructure. PN designed, synthesized and characterized the PNA. RH and FB conceived of the study and participated in its coordination. MB prepared the first draft of the manuscript and all authors contributed to its finalization and approved the final manuscript.

## Supplementary Material

Additional file 1 **Sequence of the DNA-construct**. Anchoring sequences of site A and site B of the DNA-construct and surface bound oligonucleotides as anchors. Middle part of the DNA-construct's sequence. Click here for file

Additional file 2 **Analysis of purified PNA**. (a) HPLC analysis of purified PNA 3927 (RP18 column run in 0-50% acetonitrile gradient in water, 0.1% TFA). (b) MALDI-TOF mass spectrometric analysis of purified PNA 3927. The signal from this large PNA having a molecular weight of 9856 is relative weak (and thus broad) with a center at 9863 m/e. Click here for file

Additional file 3 **Thermal denaturation profiles of PNA**. Thermal denaturation profiles of PNA 3927 hybridized to DNA oligonucleotide (a) 5'-GAG GGA AGG or (b) 5'-CAT CCA CAG GGG TAA. The experiment was performed in 100 mM NaCl, 10 mM phosphate buffer pH 7 with heating at 0.5°C/min. Click here for file
