# Supplementary material for: DNA-nanostructure-assembly by sequential spotting
Source: J Nanobiotechnology. 2011 Nov 18;9:54. doi: 10.1186/1477-3155-9-54 (PMC3248840; doi:10.1186/1477-3155-9-54)
Supplement: Additional file 1 — Sequence of the DNA-construct. Anchoring sequences of site A and site B of the DNA-construct and surface bound oligonucleotides as anchors. Middle part of the DNA-construct's sequence. [file 1477-3155-9-54-S1.PDF]

MCS of M13mp18:

```
     6231               6244     6250                   6267    6270   6274
      |                  |        |                       |       |      |
5'-acg aat tcg agc tcg gta ccc ggg gat cct cta gag tcg acc tgc agg cat gca agc ttg-3' ┐
3'-tgc tta agc tcg agc cat ggg ccc cta gga gat ctc agc tgg acg tcc gta cgt tcg aac-5' ┴ M13-mp18
                         └───────────22 bp────────────┘
```

RE-ACC65I: **RE-PstI:**

```
5'-g gtacc............ctgca g-3'
3'-ccatg g............g acgtc-5'
```

site A (Acc65I):

```
     6244
      |     ┌────────────F9───────────┐ ┌────────────F5──────────┐
5'-P-gta ccg tgg aaa gtg gca atc gtg aag gtc cgg tca taa agc gat aag-3'      M13-LF5   ┐
       3'-gc acc ttt cac cgt tag cac tt-5'                                 M13-LcF5  ┴ M13-L5
                                    3'-c cag gcc agt att tcg cta ttc-Biotin-5'   LcF5
            └───────────cF9───────────┘ └───────────cF5───────────┘
```

site B (PstI):

```
           ┌───────────cF6────────────┐ ┌───────────cF9───────────┐
5'-Biotin-ca atg aaa cac tag gcg agg ac-3'                                   RcF6
                                    5'-t tca cga ttg cca ctt tcc acc tgc a-3'   M13-RcF6 ┐
       3'-gt tac ttt gtg atc cgc tcc tga agt gct aac ggt gaa agg tgg-P-5'      M13-RF6  ┴ M13-R6
           └────────────F6────────────┘ └────────────F9───────────┘
                                                                   |
                                                                 6274
```

middle part (F10+F11):

```
                                      ┌──────────F11─────────┐
                                5'-P-cac gca agg tct gct tga ttt gga ggc tgc a-3'   M13-M2-F11  ┐
           ┌───────────F10'──────────┐         3'-c aga cga act aaa cct ccg-P-5'    M13-M2-cF11 ┴ M13-M2
5'-P-gt acc gga cga ata caa agg cta-3'            └─────cF11'─────┘                 M13-M1-F10  ┐
       3'-g cct gct tat gtt tcc gat gtg cgt tc-P-5'                                 M13-M1-cF10 ┴ M13-M1
           └───────────cF10────────────┘
```
