# Supplementary figures and images for: DNA-nanostructure-assembly by sequential spotting
Source: J Nanobiotechnology. 2011 Nov 18;9:54. doi: 10.1186/1477-3155-9-54 (PMC3248840; doi:10.1186/1477-3155-9-54)

a)

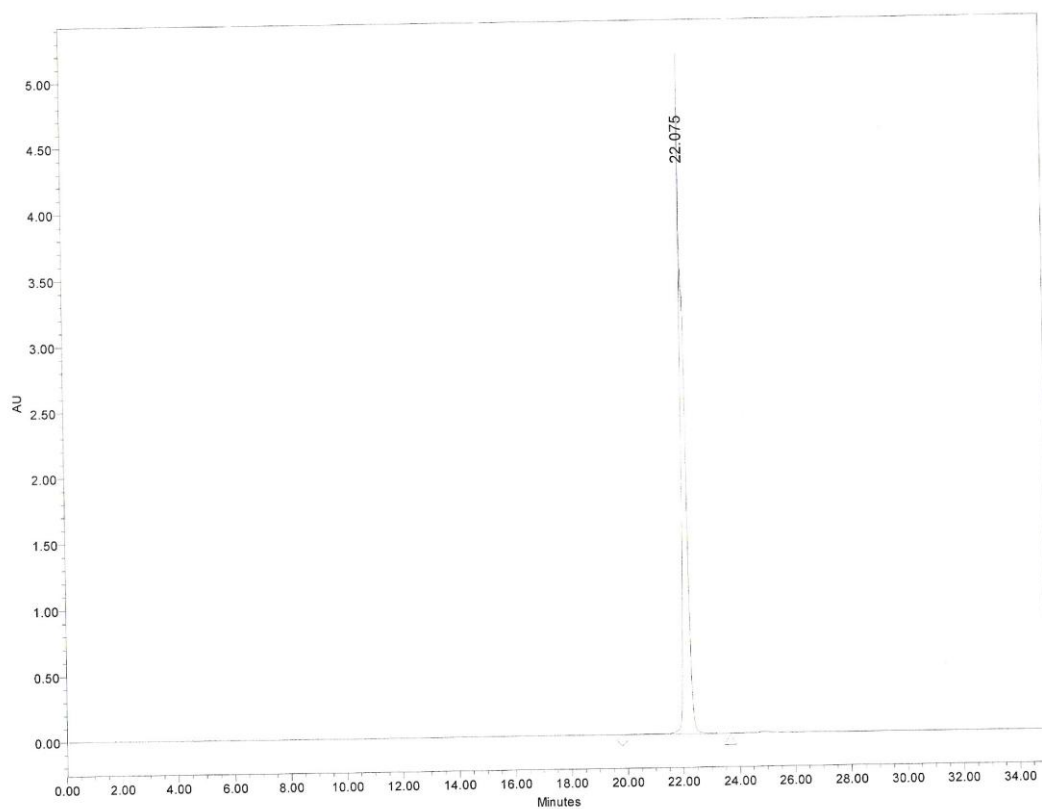

b)

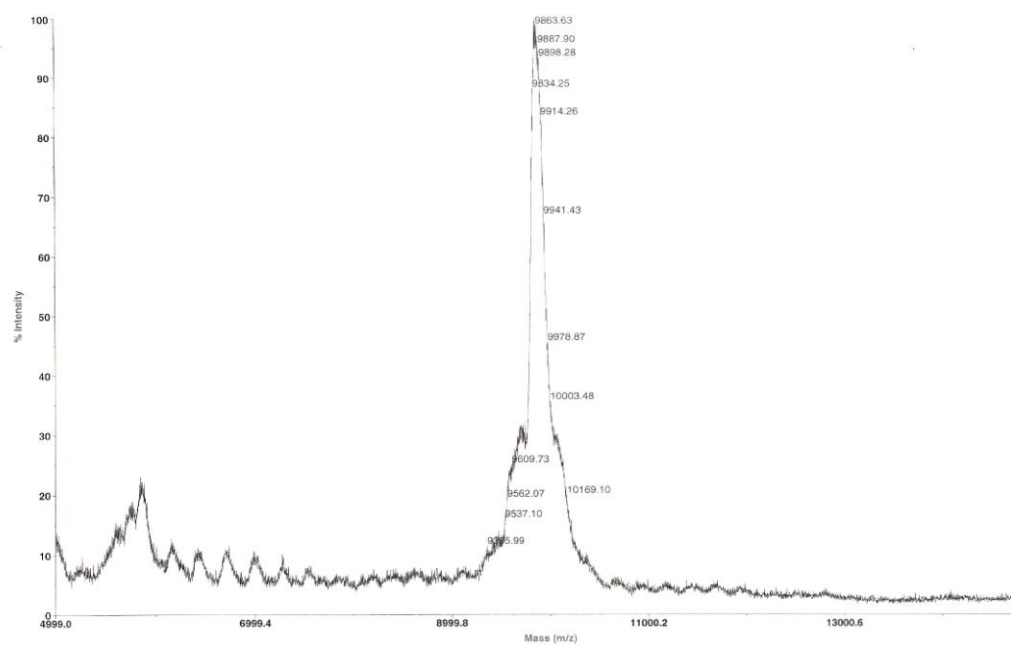

Supplement: Additional file 2 — Analysis of purified PNA. (a) HPLC analysis of purified PNA 3927 (RP18 column run in 0-50% acetonitrile gradient in water, 0.1% TFA). (b) MALDI-TOF mass spectrometric analysis of purified PNA 3927. The signal from this large PNA having a molecular weight of 9856 is relative weak (and thus broad) with a center at 9863 m/e. [file 1477-3155-9-54-S2.PDF]

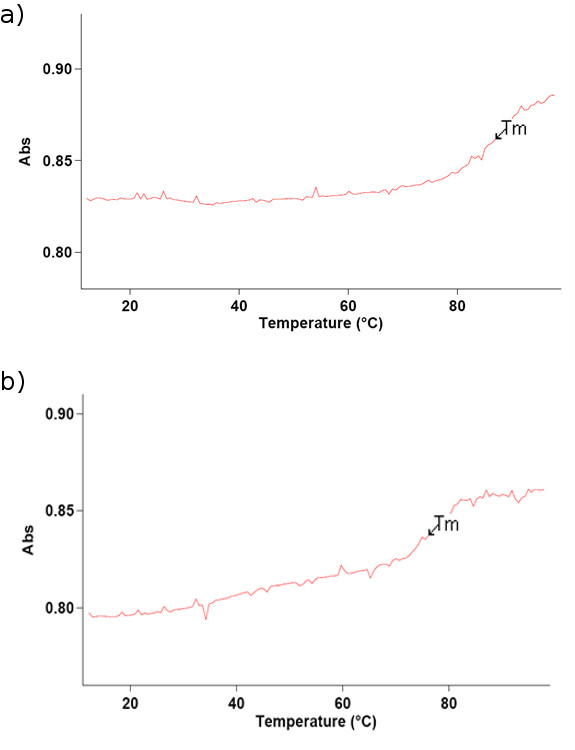

Supplement: Additional file 3 — Thermal denaturation profiles of PNA. Thermal denaturation profiles of PNA 3927 hybridized to DNA oligonucleotide (a) 5'-GAG GGA AGG or (b) 5'-CAT CCA CAG GGG TAA. The experiment was performed in 100 mM NaCl, 10 mM phosphate buffer pH 7 with heating at 0.5°C/min. [file 1477-3155-9-54-S3.JPEG]
